# Emergency Surgery during COVID-19: Lessons Learned

**DOI:** 10.1055/s-0040-1716335

**Published:** 2020-09-30

**Authors:** Hemanga K. Bhattacharjee, Shafneed Chaliyadan, Eshan Verma, Keerthi Kumaran, Priyank Bhargava, Abhishek Singh, Souvik Maitra, Rajinder Parshad

**Affiliations:** 1 Department of Surgical Disciplines, All India Institute of Medical Sciences (AIIMS), New Delhi, India; 2 Department of Anaesthesia and Critical Care, All India Institute of Medical Sciences (AIIMS), New Delhi, India

**Keywords:** COVID-19, personal protective equipment, emergency surgical procedures, operation room

## Abstract

**Introduction** The ongoing coronavirus disease-2019 (COVID-19) pandemic has disrupted health services throughout the world. It has brought in several new challenges to deal with surgical emergencies. Herein, we report two suspected cases of COVID-19 that were operated during this “lockdown” period and highlight the protocols we followed and lessons we learned from this situation.

**Result** Two patients from “red zones” for COVID-19 pandemic presented with acute abdomen, one a 64-year male, who presented with perforation peritonitis and another, a 57-year male with acute intestinal obstruction due to sigmoid volvulus. They also had associated COVID-19 symptoms. COVID-19 test could not be done at the time of their presentation to the hospital. Patients underwent emergency exploratory laparotomy assuming them to be positive for the infection. Surgical team was donned with full coverall personal protective equipment. Sudden and uncontrolled egression intraperitoneal free gas was avoided, Echelon flex 60 staplers were used to resect the volvulus without allowing the gas from the volvulus to escape; mesocolon was divided using vascular reload of the stapler, no electrosurgical devices were used to avoid the aerosolization of viral particles. Colostomy was done in both the patients. Both the patients turned out to be negative for COVID-19 subsequently and discharged from hospital in stable condition.

**Conclusion** Surgeons need to adapt to safely execute emergency surgical procedures during this period of COVID-19 pandemic. Preparedness is of paramount importance. Full precautionary measures should be taken when dealing with any suspected case.

The ongoing coronavirus disease-2019 (COVID-19) pandemic has given us new surgical challenges. Since it was first identified in Wuhan, China in December 2019, it has spread to most countries. It was declared a pandemic on March 11, 2020 by the World Health Organization (WHO). As of May 05, 2020, it has claimed the lives of more than 2.5 lakhs people and affected more than 36 lakhs people. 1 Most patients present with fever and respiratory symptoms like cough, breathlessness, and in approximately 81% disease remains mild and may not require hospitalization. 2 Minority of patients require ventilatory support and case fatality from the COVID-19 is reported between 1 and 7%. 2 About 10% of the patients may present with gastrointestinal symptoms. 3

India went into complete lockdown on March 25, 2020. 4 Many hospitals have been designated as COVID-19 centers. All elective procedures and surgeries were cancelled or postponed, so that health care facility, workers, and resources can be allocated for the care of COVID-19 patients.

Our hospital also has adopted the policy to defer all elective surgeries and only to perform emergency procedures. Emergency surgical procedures are time sensitive and require prompt decisions, but during this pandemic, the decision making becomes more difficult as the symptoms can overlap with potential SARS-CoV-2 (severe acute respiratory syndrome coronavirus-2) infection and there is a potential community spread of the infection. Waiting for COVID-19 test result leads to delay in undertaking the procedure, while immediate surgery can compromise the safety of staff and other patients in the event that the patient tests positive for infection subsequently. Other option is to consider all patients as potentially positive for the infection, unless proven otherwise.

Operating upon COVID-19 patients is both resource consuming and stressful for the surgeons. We report, herein, two patients requiring emergency surgical procedures during this lockdown period, highlighting the protocol we followed, and lessons learned from this unique situation.

## Case1

A 64-year-old male presented with complaints of abdominal pain and distention for 1 day. He had history of fever and loose stool 2 days back. He was tachycardic, tachypneic, and hypotensive on presentation. His abdomen was distended, tender, and rigid. An abdominal radiograph showed air under the diaphragm. He was resuscitated in the emergency and started on low-dose inotropic support.

He came from an area having high concentration of COVID-19 positive cases (“red zone”). Although he had no known contact with COVID-19 patients but a few of his symptoms resembled that of COVID-19. He came to the hospital in the evening; there was no provision of COVID-19 testing at that time and report could only be available by next day evening at the earliest. In view of deteriorating patient condition, the decision was taken to operate the patient as “COVID suspect” with all precautions with the assumption that the patient is COVID-19 positive.

Our institution has earmarked wards and operation room (OR) for COVID-19 suspected patients. The designated OR area for COVID-19 suspected patients has anterooms for donning (unsterile and sterile areas) and doffing. Patient was brought from the designated ward to the OR through a separate corridor and an elevator, which was earmarked for the transport of “COVID-19 suspect” patients. The corridor through which the patient would enter and exit OR was also predefined and was separate from that of the hospital staff. Patient was accompanied by a hospital assistant and a physician wearing coverall personal protective equipment (PPE). The operating team entered through a different corridor of the OR and team had only minimum number of staffs. The detailed steps for donning with PPE with coverall gowns, N95 mask, eye wear, face shield, gum boots, shoe covers, were illustrated on an OT board and were supervised by a senior nursing staff. After donning, identification of the patient was performed by the surgeon who had earlier seen the patient in the emergency department. Anesthetists intubated the patient while rest of the team remained outside. A rapid sequence induction technique was used without any mask ventilation to reduce aerosol generation. Senior most member of the anesthesia team performed intubation by a video laryngoscope to reduce intubation time. Surgeons and scrub nurse did hand hygiene using alcohol-based solution on already donned “coverall” PPE and wore impervious sterile gown and gloves in the sterile donning area. Once the anesthesia team gave go ahead, the fully donned surgeons and nursing staff (a scrub nurse and a floor nurse) entered the OT and prepared the patient.

A midline incision was made, extended deep and peritoneum was opened slowly using scalpel. Free peritoneal gas and contaminated fluid were readily sucked from the operating field using two sets of suction apparatus. A large slough out area with perforation was identified in the sigmoid colon. Bowel above and below the perforation was unhealthy. Sigmoid colon was transected using Echelon Flex Endopath staplers (Ethicon Endo-Surgery, LLC, PR) above and below the perforated segment and the segment was excised. To avoid aerosol generation electrosurgery devices were not used. Sigmoid mesocolon was divided using a vascular reload of the stapler (white cartridge). Proximal end of the colon was brought out as stoma and stoma bag applied immediately. Patient was not extubated inside the OR and shifted to the designated COVID-19 suspect ward. The surgical team did doffing under supervision of an experienced staff in the designated area and took shower before coming out of OR complex. The patient was shifted to general ward on the next day evening after his COVID-19 test report came out as negative. In view of his persistent tachypnea, CECT thorax was done on postoperative day two, which revealed pulmonary embolism and was managed conservatively. Patient was discharged from hospital on 12th postoperative day.

## Case 2

A 57-year-old male, presented with abdominal distension, nausea, and vomiting for 7 days. He had obstipation since past 5 days. He was a known patient of bronchial asthma, hypertension, and vascular dementia. His pulse was 96/min, blood pressure was 110/60 mm Hg, and respiratory rate was 26/min. The abdomen was distended, tender with features of peritonism. There were bilateral infra-axillary crepitations on chest auscultation. His abdominal plain radiograph showed features of sigmoid volvulus and chest radiograph showed right lower zone opacities. CECT abdomen and thorax confirmed those findings. Although he had no history of contact with COVID-19 patient, he came from a “red zone” area and a few of his symptoms resembled that of COVID-19. COVID-19 testing could not be done at the time of presentation. He was taken up to OR as a “COVID suspect” assuming a positive status. Exploratory laparotomy was performed. There was a sigmoid volvulus with dusky sigmoid colon. Volvulus was derotated; colon was transected proximal and distal to the volvulus using Echelon Flex Endopath staplers. Sigmoid mesocolon was divided with vascular reloads of the stapler (white cartridges). Proximal end was brought out of the abdomen as stoma and stoma bag was applied immediately. No energy devices were used during the entire procedure. Patient was not extubated inside the OR and was shifted to COVID suspect ward. He was transferred to general ward after his COVID-19 test report came negative on the next day. He made an uneventful recovery and was discharged on eighth postoperative day.

## Discussion

COVID-19 pandemic has brought in several new challenges to deal with surgical emergencies. International and national societies have published guidelines to address this situation. 5 6 7 8 9 However, local situation may vary from hospital to hospital and health care personnel need to adapt and improvise to deliver safe and effective health care. Waiting period for confirmatory test may take from 24 hours to 5 days. 9 10 Our hospital has in-house severe acute respiratory syndrome coronavirus-2 (SARS-CoV-2) reverse transcription polymerase chain reaction testing and performs it twice a day, once in the morning 10.00 am and the other at 3:00 pm. Reports are usually available by approximately 7 hours. All positive cases are sent to another designated building for further care. Emergency surgical patients who come during odd hours of the day pose a special concern.

Both of our patients came in afternoon hours and required urgent surgical intervention. They came from high alert area of COVID-19. The Government of India has divided country into zones based on the number of cases, the recovery rate, the doubling rate, etc. 11 Red zone districts account for more than 80% cases in the state or have a doubling rate of less than 4 days. Patients had few suspicious features of COVID-19 making them “COVID-19 suspects.” Waiting for confirmatory test result would have delayed interventions by at least 24 hours. So, we decided not to wait for results and performed the procedures assuming patients are COVID-19 positive.

In both the cases, our surgical target region was the gastrointestinal tract. Presence of SARS-CoV-2 ribonucleic acid virus has been detected in enteric content, peritoneal fluid, and feces of the positive patients. 12 13 It has been shown that virus was detectable in stool of positive patients regardless of the severity of illness and for a longer duration than any other body fluids. 13 In the first case, there was free intraperitoneal gas, which may be infectious in nature. To reduce the sudden and uncontrolled egression of free intraperitoneal gas, we made a small hole on the peritoneum and one suction canula was inserted to the peritoneal cavity and the other kept around the entry. This helped us in gradual evacuation of intraperitoneal contents. A closed system with attached filters would have been ideal for the evacuation. In absence of that, we kept the suction apparatus, which collected the contents far away as possible from the health care worker. In our second case, the sigmoid colon was hugely distended and tensed with gas. Application of staplers to divide the colon proximal and distal to the volvulus helped us to minimize any escape of colonic gas and to deliver the volvulus intact.

Electrosurgical devices used during surgery have been found to be associated with the production of aerosolized viral particles in some blood–borne viruses. 14 15 16 As such, with the use of electrocautery, surgery becomes an “aerosol generating procedure.” 17 Due to the potential infectious nature of this aerosol, use of electrosurgical devices (monopolar electrocautery or ultrasonic device) has been cautioned in COVID-19 patients. 18 A monopolar device when needs to be used, should be kept in lowest energy setting and to be used in combination with a smoke evacuation system. 18 19 However, we did not have a smoke evacuation system in our OR. Although, the monopolar electrocautery was kept standby, we were able to completely avoid their use. In both cases, dissection and tissue division were done using scissors and blade. Hemostasis was achieved by suture ligation or warm compression.

We used vascular stapler to divide the mesocolon. Due to their regular application in our non-COVID-19 practice, our team was well versed with their application. We believe and have experienced that, operating with full personal protective gear causes immense physical and mental stress. Disturbances caused by the mist on the goggles and face shield adds to the misery. All measures should be taken to execute surgery safely without any undue prolongation.

The indication and type of emergency procedure may also vary in pandemic. Conditions where nonoperative management is feasible should be practiced. These include conservative management in conditions like uncomplicated appendicitis, acute cholecystitis, and diverticulitis. 8 However, some authors have opined that for hospitals with limited resources, emergency appendicectomy might be favored over conservative management, 20 as the former is associated with earlier hospital discharge. 21 This can potentially save the health care resources that could be used for the ongoing pandemic.

There are differences of opinion in regard to certain clinical practices. The Spanish Society of Surgeons guidelines recommend caution on creating stoma, as it is a possible focus of transmission of infection to hospital personnel and patient relatives. 7 The Italian group on colorectal surgery has, however, recommended Hartmann's procedure over anastomosis, because the latter in emergency setting is associated with higher risk of complications (e.g., anastomotic leak, intra-abdominal collection), that can result in consumption of more hospital resources and can be complicated further by co-existing COVID-19 infection status. 13 In our first case, the patient merited a stoma. In the second case, anastomosis was also a possibility, but we chose to perform stoma to reduce the possible postoperative complications.

Another contentious issue is the use of laparoscopy during COVID-19 pandemic. Due to pneumoperitoneum and low gas movement, aerosols created during laparoscopic surgery tend to get concentrated inside the abdomen. During the release of trocar valves or nonair-tight exchange of instrument, there is sudden egression of aerosols through these ports. Due to these potential risks of increased aerosolization and possible viral transmission to health care workers, laparoscopic approach is discouraged. 19 22 On the other hand, laparoscopic surgery in most cases is associated with early recovery, reduced hospital stay, and reduction in complications minimizing the consumption of hospital resources that have already been strained due to the ongoing pandemic. Furthermore, other advantages of laparoscopic approach such as inherent physical barrier between patient and surgical team and more controlled and safer way of releasing surgical smoke and aerosol make laparoscopic approach an attractive option in pandemic setting. The Society of American Gastrointestinal and Endoscopic Surgeons (SAGES) and European Association of Endoscopic Surgery (EAES) guidelines therefore recommend laparoscopy, with strong suggestion to consider the use of devices to filter released CO _2_ for aerosolized particles. 6

## Conclusion

Surgeons might need to modify their approaches even for common surgical procedures during the pandemic so that timely intervention can be done. Careful planning and logistical preparedness to address such unprecedented situation are of paramount importance for safety of the patients as well as the health care worker.
